# Minimally invasive versus open emergency right-sided colectomy for malignant obstruction: a nationwide propensity score–matched cohort study

**DOI:** 10.1186/s13017-026-00735-6

**Published:** 2026-10-06

**Authors:** Erik Agger, Peter Matthiessen, Tomas Vedin

**Affiliations:** 1 Department of Surgery, Skåne University Hospital, Malmö, SE-214 21 Sweden; 2 Department of Clinical Sciences, Lund University, Lund, Sweden; 3 Department of Surgery, Örebro University Hospital, Örebro, Sweden; 4 Department of Surgery, Faculty of Medicine and Health Sciences, Örebro University, Örebro, Sweden

**Keywords:** Minimally invasive surgery, Colon cancer, Bowel obstruction, Emergency surgery, Propensity score matching

## Abstract

**Background:**

Minimally invasive surgery (MIS) improves short-term outcomes in elective colectomy, however, the choice of surgical approach in hemodynamically stable patients with obstructing right-sided colon cancer remains unclear in emergency settings. MIS could be considered and the present study aims to evaluate short-term outcomes of MIS in bowel obstructed patients compared with open surgery (OPEN).

**Methods:**

A retrospective nationwide study including patients from the nationwide Swedish Colorectal Cancer Registry (SCRCR) undergoing emergency right-sided colectomy in Sweden (2017–2023) was conducted. Propensity score matching (1:1) was performed based on sex, age, ASA class, hospital size, and operative timing (on-call vs. daytime). Outcomes included length of stay (LOS), postoperative complications, and surgical quality indicators. Analysis was performed according to intention-to-treat.

**Results:**

A total of 1147 patients were identified. Of the 1,147 included patients, 99 (8.6%) underwent an initially minimally invasive approach. After matching, 99 patients remained in each group. The conversion rate was 34%.

Median LOS was 9 days (IQR 6;14) after OPEN and 6 days (IQR 5;10) after MIS. In quantile regression with bootstrap standard errors, MIS was associated with a 3-day shorter median LOS (median difference − 3.0 days, 95% CI − 4 to − 2, *p* = 0.002).

Postoperative complications (Clavien-Dindo ≥ 2) occurred in 38.8% after OPEN and in 23.2% after MIS. Logistic regression analysis with robust standard errors showed significantly lower odds of postoperative complications after MIS (OR 0.48, 95% CI 0.27–0.83, *p* = 0.009).

No significant differences were observed in re-admission, 90-day mortality, R0 resection, lymph node yield, blood loss or operative time.

**Conclusion:**

In this nationwide observational cohort, an initially minimally invasive approach was associated with shorter length of stay and fewer postoperative complications. No statistically significant differences were observed in the available short-term surgical and pathological quality indicators. Given the limited number of MIS procedures and potential for residual confounding, these findings support further evaluation of MIS in carefully selected patients.

**Supplementary Information:**

The online version contains supplementary material available at https://doi.org/10.1186/s13017-026-00735-6.

## Key points

- MIS was associated with reduced LOS by 3 days in emergency right-sided colectomy.

- MIS was associated with lower complication rates.

- No statistically significant differences were observed in measured short-term surgical and pathological quality indicators.

- Findings support selective use in emergency settings.

**Supplementary Information:**

The online version contains supplementary material available at https://doi.org/10.1186/s13017-026-00735-6.

## Lay summary

**Background:**

In planned colon cancer surgery keyhole (minimally invasive) surgery is the most common approach. In emergency situations, however, open surgery with a midline incision is still the most common approach. There are several known benefits to patients undergoing keyhole surgery such as less pain, shorter length of hospital stay and decreased risk of incisional hernia.

**Aim:**

This study investigated whether keyhole surgery provides benefits in emergency operations for bowel obstruction caused by cancer, and whether it is safe.

**Method:**

Data from the nationwide Swedish Colorectal Cancer Registry (SCRCR) 2017-2023 were analyzed.

**Findings and conclusion:**

Patients who underwent keyhole surgery had a shorter hospital stay and fewer complications. No clear differences were identified in the measured short-term surgical and pathological indicators. These findings suggest that keyhole surgery may be a feasible option for carefully selected patients requiring emergency surgery.

## Introduction

Minimally invasive surgery (MIS) is established as the preferred approach in elective colorectal cancer surgery owing to improved short-term outcomes without compromising oncological safety [1–3]. Although minimally invasive surgery has become the standard approach for elective colorectal cancer resection because of faster recovery and reduced postoperative morbidity, its adoption in emergency colorectal surgery has been considerably slower. Concerns regarding bowel distension, advanced tumour stage, technical complexity, longer duration of surgery and the need for urgent intervention have limited widespread implementation. Recent retrospective studies have suggested that laparoscopic emergency right-sided colectomy may be feasible and associated with shorter hospital stay while maintaining acceptable oncological outcomes. However, existing evidence is predominantly derived from single-centre studies and selected patient cohorts, leaving uncertainty regarding the generalisability of these findings at a national level [4, 5].

Malignant obstruction occurs in approximately 8–13% of patients with colon cancer and is associated with increased morbidity, mortality and impaired oncological outcomes compared with elective treatment. Emergency surgery therefore remains a particularly demanding clinical scenario requiring rapid decision-making while balancing immediate patient safety with long-term oncological principles. In contrast to left-sided obstruction, decompressive alternatives such as defunctioning stomas and self-expanding metal stents are less established for right-sided disease, and current international guidelines recommend right-sided colectomy as the preferred treatment in most cases [6–9].

Recent studies suggest that MIS may be feasible in emergency colorectal surgery, but high-quality population-based data are lacking [10–13]. Furthermore, studies suggest that minimally invasive emergency right-sided colectomy is feasible and may offer improved postoperative recovery without compromising surgical quality [4]. Nevertheless, available evidence remains limited by small sample sizes, single-centre designs and substantial risk of selection bias. High-quality population-based data are lacking. Therefore, the aim of the present nationwide multicentre study was to evaluate short-term outcomes following emergency right-sided colectomy for malignant obstruction using data from the SCRCR [14]. We hypothesized that MIS would be associated with shorter length of stay (LOS) and fewer postoperative complications while maintaining comparable short-term surgical and pathological quality indicators.

## Methods

### Study design and population

This was a retrospective nationwide cohort study including patients in Sweden who underwent emergency right-sided colectomy between 1 January 2017 and 31 December 2023 due to malignant obstruction (ICD-10 codes C18.0, C18.2, C18.3, C18.4). Patients were identified through the SCRCR. All tumour stages were eligible, provided a bowel resection was completed.

**Fig. 1:**
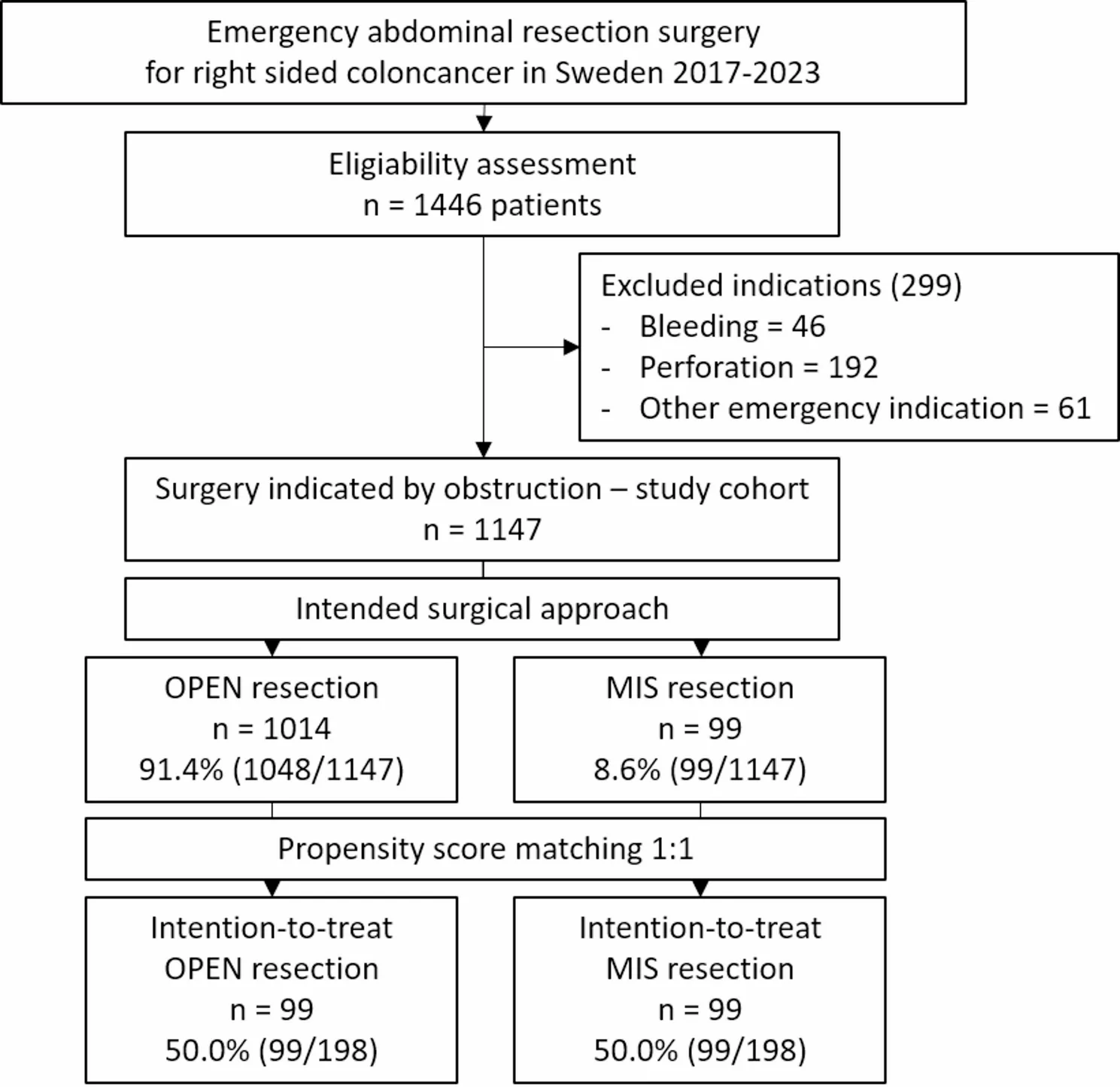
Flow diagram of patient selection. Flow diagram showing inclusion and exclusion of patients undergoing emergency right-sided colectomy for malignant obstruction. MIS, minimally invasive surgery

The study was designed to evaluate short-term outcomes following minimally invasive surgery (MIS) compared with open resection (OPEN) according to intention-to-treat. Patients undergoing emergency surgery due to tumour bleeding, perforated colon cancer, or other indications were excluded. Details of patient selection are presented in Fig. 1.

The SCRCR has a coverage rate of > 99% and has demonstrated high internal and external validity for variables related to patient demographics, surgical procedures, postoperative complications, and histopathology [15, 16].

The choice of surgical approach (MIS or OPEN) was at the discretion of the treating surgeon according to local practice and clinical assessment. Detailed information regarding radiological severity of obstruction, bowel dilatation, duration of symptoms, haemodynamic parameters, and other factors influencing operative decision-making was not available within the registry.

This study is reported in accordance with the STROBE guidelines for observational studies.

### Outcomes

The primary outcomes length of stay (LOS) and postoperative complications defined as Clavien–Dindo grade ≥ 2 were prespecified co-primary outcomes. No formal multiplicity adjustment was applied and secondary outcomes including 90-day mortality, reoperation, readmission, R0 resection rate, lymph node yield, intraoperative blood loss, and operative time were considered exploratory.

### Statistical analysis

Patients undergoing OPEN and MIS were matched using propensity score matching (PSM). The propensity score was estimated using logistic regression including sex, age, ASA class, hospital size, and operative timing (on-call versus daytime), variables considered to influence the choice of surgical approach. Matching was performed using 1:1 nearest-neighbour matching without replacement and a caliper of 0.1 standard deviations of the logit of the propensity score. Balance between groups was assessed using standardized mean differences (SMD), with values < 0.1 indicating good balance [17]. Supplementary Fig. 1 shows covariate balance before and after matching.

Variables included in the propensity score model were selected a priori based on clinical relevance and availability within the SCRCR. Age, sex, ASA class, hospital size, and timing of surgery were considered potential determinants of both treatment selection and postoperative outcomes. Several potentially important confounders, including physiological status at presentation, radiological severity of obstruction, bowel dilatation, duration of symptoms, tumour complexity, surgeon experience and institutional MIS volume, were not available in the registry and could therefore not be included in the matching procedure. Patients with missing values in variables included in the propensity score model were excluded from matching.

Categorical variables are reported as counts and percentages. Continuous variables are presented as mean (standard deviation) or median (interquartile range, IQR), depending on distribution. Group comparisons were performed using Pearson’s χ² test for categorical variables, Student’s t-test for normally distributed continuous variables, and the Mann–Whitney U test for non-normally distributed variables.

The co-primary outcomes were postoperative complications and length of hospital stay.

Binary outcomes (postoperative complications, multivisceral resection, stoma formation, R0 resection, 30-day readmission, and 90-day mortality) were analyzed separately using logistic regression. To account for the dependence between patients within matched pairs, cluster-robust standard errors were calculated with the matched pair as the clustering unit. Results were reported as odds ratios (ORs) for MIS compared with open surgery, with corresponding 95% confidence intervals (CIs) and two-sided p-values. Stoma formation was defined as the construction of either a permanent or a diverting stoma. R0 resection was analyzed as R0 versus R1/R2 resection.

Length of hospital stay, number of examined lymph nodes, intraoperative blood loss, and operative time were analyzed using median quantile regression. The regression coefficient represents the median difference between the MIS and open groups, expressed as MIS minus open surgery. Bootstrap standard errors and 95% percentile CIs were obtained from 5,000 non-parametric bootstrap samples. To preserve the matched design, resampling was performed at the matched-pair level, with both members of each sampled pair retained in every bootstrap sample. For the quantile regression analyses, two-sided p-values were derived from the tails of the same bootstrap distributions generated by resampling entire matched pairs.

Analyses were based on the available observations for each outcome, without imputation of missing outcome data. The remaining safety and oncological outcomes were considered secondary exploratory outcomes and were interpreted using effect estimates and their 95% CIs.

All statistical tests were two-sided, and a p-value < 0.05 was considered statistically significant. Analyses were performed using SPSS (IBM SPSS Statistics version 29.0.2.0, IBM Corp., Armonk, NY, USA) and R Statistical Software (version 4.5.1; R Core Team 2025).

### Definitions

Emergency resection was defined as non-elective surgery performed during the index admission due to obstruction requiring urgent intervention.

Length of stay was calculated from the date of surgery until discharge from hospital.

Postoperative complications were graded according to the Clavien–Dindo classification [18]. The SCRCR records complications of grade 2–5, and these were used to define complication rates [19]. Postoperative complications are recorded within 30 days of surgery in the Swedish Colorectal Cancer Registry. Complications identified after discharge, including during readmission within 30 days, were captured according to the registry’s standard reporting procedures.

Intraoperative blood loss was recorded as the estimated blood loss documented at the completion of surgery by the anaesthesia team and surgical staff as part of routine perioperative reporting. No standardized gravimetric measurement protocol was used.

### Ethical considerations

The study was approved by the Swedish Ethical Review Authority (DNR: 2024-02208-01) and the SCRCR steering committee. Registration of colorectal cancer in the SCRCR is mandatory in Sweden, with the option for individual patients to opt out. The study was retrospective, and no interventions to treatment or follow-up were performed.

## Results

### Study population

In total, 1446 patients underwent right-sided colectomy between January 1st 2017 and December 31st 2023 and were identified for eligibility assessment. Surgery was indicated by obstruction among 1147 patients and thus met the study inclusion criteria, while 299 patients were excluded due to other indications for emergent resection (Fig. 1). The most common reason for exclusion was bowel perforation. Patients were operated across a total of 46 centres; 23 performed both OPEN and MIS; 15 only OPEN; 8 only MIS in the matched cohort (Supplement 2).

In total, 99 patients (8.6%) underwent an initially minimally invasive approach, of whom 34 (34%) were converted to open surgery (Fig. 1).

Baseline patient characteristics before and after matching are summarized in Table 1. After matching, SMD between groups regarding sex, age, ASA class, hospital size, and operative timing (on-call vs. daytime) was below the accepted standardized mean difference (SMD) threshold (< 0.1). Tumours were located in the caecum, ascending colon, hepatic flexure, and transverse colon. Although no statistically significant difference in tumour location was observed between groups after matching, the greatest numerical difference was seen for caecal tumours, which were more common in the MIS group (48.5% vs. 38.4%), while hepatic flexure and transverse colon tumours were numerically more frequent in the OPEN group. Multivisceral resections were performed to a similar extent in the matched OPEN and MIS group (14.1% vs. 11.1%, *p* = 0.521); Table 1. Permanent ostomy creation was similar between groups at 9.1% for OPEN compared to 12.1% after MIS-resection (*p* = 0.489); Table 2.

**Table 1:** Baseline characteristics in unmatched and intention-to-treat matched cohorts

Table 1 - Baseline patient characteristics		Unmatched cohort - intention to treat				Matched cohort - intention to treat			
		All patients	Open	MIS		All patients	Open	MIS	
Patients		1147	1048 (91.4)	99 (8.6)	SMD	198 (100)	99 (50.0)	99 (50.0)	SMD
Sex	Male	486 (42.4)	446 (42.6)	40 (40.4)	-0.04	78 (39.4)	38 (38.4)	40 (40.4)	0.04
	Female	661 (57.6)	602 (57.4)	59 (59.6)		120 (60.6)	61 (61.6)	59 (59.6)	
Age	Mean (± SD)	75.0 (12.3)	75.0 (12.4)	74.7 (11.2)	0.02	74.6 (10.9)	74.6 (10.6)	74.7 (11.2)	-0.01
Age group	20–39	15 (1.3)	15 (1.4)	0	0.07	0	0	0	0.05
	40–59	120 (10.5)	109 (10.4)	11 (11.1)		20 (10.1)	9 (9.1)	11 (11.1)	
	60–79	553 (48.2)	498 (47.5)	55 (55.6)		111 (56.1)	56 (56.6)	55 (55.6)	
	80–100	459 (40.0)	426 (40.6)	33 (33.3)		67 (33.8)	34 (34.3)	33 (33.3)	
	Missing	0	0	0		0	0	0	
BMI	Mean (± SD)	24.7 (4.7)	24.7 (4.7)	24.6 (4.9)	0.03	25.1 (5.1)	25.6 (5.3)	24.6 (4.9)	0.19
	Missing	166	152	14		23	9	14	
ASA-class	1	82 (7.1)	78 (7.4)	4 (4.0)	0.04	8 (4.0)	4 (4.0)	4 (4.0)	0.00
	2	434 (37.8)	389 (37.1)	45 (45.5)		89 (44.9)	44 (44.4)	45 (45.5)	
	3	520 (45.3)	475 (45.3)	45 (45.5)		92 (46.5)	47 (47.5)	45 (45.5)	
	4	84 (7.3)	79 (7.5)	5 (5.1)		9 (4.5)	4 (4.0)	5 (5.1)	
	5	2 (0.2)	2 (0.2)	0		0	0	0	
	Missing	25 (2.2)	25 (2.4)	0		0	0	0	
On call hours	No	718 (62.6)	634 (60.5)	84 (84.8)	0.51	168 (84.8)	84 (84.8)	84 (84.8)	0.00
	Yes	429 (37.4)	414 (39.5)	15 (15.2)		30 (15.2)	15 (15.2)	15 (15.2)	
	Missing	0	0	0		0	0	0	
Hospital size	Small (0–99)	42 (3.7)	31 (3.0)	11 (11.1)	0.43	22 (11.1)	11 (11.1)	11 (11.1)	0.02
(beds)	Mid size (100–249)	159 (13.9)	138 (13.2)	21 (21.2)		41 (20.7)	20 (20.2)	21 (21.2)	
	Large (250–499)	405 (35.3)	375 (35.8)	30 (30.3)		60 (30.3)	30 (30.3)	30 (30.3)	
	Very large (500+)	541 (47.2)	504 (48.1)	37 (37.4)		75 (37.9)	38 (38.4)	37 (37.4)	
	Missing	0	0	0		0	0	0	
Pre-OP CT-stage	No	189 (16.5)	176 (16.8)	13 (13.1)	-0.10	23 (11.6)	10 (10.1)	13 (13.1)	0.09
	Yes	951 (82.9)	865 (82.5)	86 (86.9)		175 (88.4)	89 (89.9)	86 (86.9)	
	Missing	7 (0.6)	7 (0.7)	0		0	0	0	
Tumour location	Appendix	9 (0.8)	8 (0.8)	1 (1.0)	0.30	1 (0.5)	0	1 (1.0)	0.25
in colon	Caecum	440 (38.4)	392 (37.4)	48 (48.5)		86 (43.4)	38 (38.4)	48 (48.5)	
	Ascending colon	292 (25.5)	264 (25.2)	28 (28.3)		55 (27.8)	27 (27.3)	28 (28.3)	
	Hepatic flexure	160 (13.9)	151 (14.4)	9 (9.1)		27 (13.6)	18 (18.2)	9 (9.1)	
	Transverse colon	246 (21.4)	233 (22.2)	13 (13.1)		29 (14.6)	16 (16.2)	13 (13.1)	
	Missing	0	0	0		0	0	0	
Surgery performed	Ileoceacal res	30 (2.6)	26 (2.5)	4 (4.0)	0.10	5 (2.5)	1 (1.0)	4 (4.0)	0.19
	Right hemi	1117 (97.4)	1022 (97.5)	95 (96.0)		193 (97.5)	98 (99.0)	95 (96.0)	
	Missing	0	0	0		0	0	0	
Multivisceral	No	989 (86.2)	901 (86.0)	88 (88.9)	0.08	173 (87.4)	85 (85.9)	88 (88.9)	0.09
resection	Yes	158 (13.8)	147 (14.0)	11 (11.1)		25 (12.6)	14 (14.1)	11 (11.1)	
	Missing	0	0	0		0	0	0	
pT-stage	T1-2	54 (4.7)	44 (4.2)	10 (10.1)	0.11	14 (7.1)	4 (4.0)	10 (10.1)	0.17
	T3	460 (40.1)	425 (40.6)	35 (35.4)		71 (35.9)	36 (36.4)	35 (35.4)	
	T4	601 (52.4)	549 (52.4)	52 (52.5)		107 (54.0)	55 (55.6)	52 (52.5)	
	Missing	32 (2.8)	30 (2.9)	2 (2.0)		6 (3.0)	4 (4.0)	2 (2.0)	
M-stage	M0	863 (75.2)	787 (75.1)	76 (76.8)	-0.01	147 (74.2)	71 (71.7)	76 (76.8)	0.07
	M1	213 (18.6)	194 (18.5)	19 (19.2)		40 (20.2)	21 (21.2)	19 (19.2)	
	Missing	71 (6.2)	67 (6.4)	4 (4.0)		11 (5.6)	7 (7.1)	4 (4.0)	
Final tumour stage	I	43 (3.7)	35 (3.3)	8 (8.1)	0.17	11 (5.6)	3 (3.0)	8 (8.1)	0.30
	II	240 (20.9)	221 (21.1)	19 (19.2)		33 (16.7)	14 (14.1)	19 (19.2)	
	III	427 (37.2)	395 (37.7)	32 (32.3)		72 (36.4)	40 (40.4)	32 (32.3)	
	IV	281 (24.5)	260 (24.8)	21 (21.2)		48 (24.2)	27 (27.3)	21 (21.2)	
	Missing	156 (13.6)	137 (13.1)	19 (19.2)		34 (17.2)	15 (15.2)	19 (19.2)	

Values are *n* (%) unless otherwise indicated. Continuous variables are presented as mean (SD). Balance was assessed using standardized mean differences (SMD)

*BMI* body mass index, *ASA* American Society of Anesthesiologists

**Table 2:** Perioperative surgical and short-term pathological outcomes in the matched cohort

Patients		All patients	OPEN	MIS
		198 (100)	99 (50.0)	99 (50.0)
Re-operation	No	180 (90.9)	88 (88.9)	92 (92.9)
	Yes	15 (7.6)	10 (10.1)	5 (5.1)
	Missing	3 (1.5)	1 (1.0)	2 (2.0)
Number of	0	180 (90.9)	88 (88.9)	92 (0.9)
re-operations	1	13 (6.6)	8 (8.1)	5 (5.1)
	2 or more	2 (1.0)	2 (2.0)	0
	Missing	3 (1.5)	1 (1.0)	2 (2.0)
Re-admission	No	177 (89.4)	90 (90.9)	87 (87.9)
	Yes	18 (9.1)	8 (8.1)	10 (10.1)
	Missing	3 (1.5)	1 (1.0)	2 (2.0)
Postoperative	No	133 (67.2)	60 (60.6)	73 (73.7)
complication (any)	Yes	60 (30.3)	38 (38.4)	22 (22.2)
	Missing	5 (2.5)	1 (1.0)	4 (4.0)
Death within 90 days	Yes	12 (6.1)	7 (7.1)	5 (5.1)
Surgical radicality	R0	176 (88.9)	86 (86.9)	90 (90.9)
	R1	4 (2.0)	2 (2.0)	2 (2.0)
	R2	11 (5.6)	7 (7.1)	4 (4.0)
	Missing	7 (3.5)	4 (4.0)	3 (3.0)
Tumour perforation	No	187 (94.4)	92 (92.9)	95 (96.0)
during surgery	Yes	9 (4.5)	6 (6.1)	3 (3.0)
	Missing	2 (1.0)	1 (1.0)	1 (1.0)
Lymph nodes yield	nr (median, IQR)	26 (18–35)	27 (19–35)	23 (17–34)
	Missing	7	4	3
Bleeding during	mL (median, IQR)	50 (10–150)	100 (25–150)	50 (10–100)
surgery	Missing	10	6	4
Duration of surgery	min (mean ± SD)	170 (64)	170 (65)	170 (63)
	Missing	2	2	0
Permanent ostomy	No	177 (89.4)	90 (90.9)	87 (87.9)
	Yes	21 (10.6)	9 (9.1)	12 (12.1)
	Missing	0	0	0

Values are *n* (%) unless otherwise indicated. Percentages are based on available observations for each variable. Missing data are reported separately where applicable. Continuous variables are presented as median (IQR) unless stated otherwise

*IQR* interquartile range, *SD* standard deviation

### Length of stay

The median LOS was shorter in the MIS group at 6 (IQR 5–10) days compared to patients in the OPEN group who were discharged after a median of 9 (IQR 6–14) days. In quantile regression with bootstrap standard errors, MIS was associated with a 3-day shorter median LOS (median difference − 3.0 days, 95% CI − 4 to − 2, *p* = 0.002); Table 3.

**Table 3:** Effect estimates for binary and continuous outcomes in the matched intention-to-treat cohort

Outcome	Open surgery	MIS	Effect estimate for MIS start vs. open surgery (95% CI)	*P* value
Binary outcomes, n/N (%)				
Postoperative complications*	38/98 (38.8%)	22/95 (23.2%)	OR 0.48 (0.27–0.83)	0.009
30-day readmission	8/98 (8.2%)	10/97 (10.3%)	OR 1.29 (0.49–3.42)	0.605
90-day mortality	7/99 (7.1%)	5/99 (5.1%)	OR 0.70 (0.23–2.15)	0.532
R0 resection	86/95 (90.5%)	90/96 (93.8%)	OR 1.57 (0.60–4.12)	0.360
Stoma formation	9/99 (9.1%)	12/99 (12.1%)	OR 1.38 (0.55–3.48)	0.495
Continuous outcomes, median (IQR)				
Length of hospital stay, days*	9 (6–14); *n* = 97	6 (5–10); *n* = 96	MD -3 (-4 to -2)	0.002
Examined lymph nodes, n	27 (19–35); *n* = 95	23 (17-33.3); *n* = 96	MD -4 (-7 to 0)	0.143
Intraoperative blood loss, mL	100 (25–150); *n* = 93	50 (10–100); *n* = 95	MD -50 (-75 to 0)	0.206
Duration of surgery, minutes	159 (126–201); *n* = 97	165 (125.5-201.5); *n* = 99	MD 6 (-12 to 26)	0.456

Values are presented as n/N (%) or median (interquartile range). Complete case analysis was performed. Odds ratios and corresponding 95% confidence intervals were estimated using logistic regression with cluster-robust standard errors at the matched-pair level. Median differences and percentile confidence intervals were estimated using median quantile regression with 5,000 bootstrap samples, resampling entire matched pairs. Median differences (MD) are expressed as MIS minus open surgery; negative values indicate lower values in the MIS group. P-values are reported for the logistic regression analyses. For the quantile regression analyses, two-sided p-values were derived from the tails of the same bootstrap distributions generated by resampling entire matched pairs

*CI* confidence interval, *IQR* interquartile range, *MIS* minimally invasive surgery, *OR* odds ratio

*Co-primary outcome

### Postoperative complications

Postoperative complications occurred in 38.8% (*n* = 38/98) of patients in the OPEN group and 23.2% (*n* = 22/95) of patients in the MIS group with reported values. Logistic regression analysis with robust standard errors showed significantly lower odds of postoperative complications after MIS (OR 0.48, 95% CI 0.27–0.83, *p* = 0.009).

Readmission within 30 days occurred in 8 patients (8.2%) in the OPEN group and 10 patients (10.3%) in the MIS group (OR 1.29, 95% CI 0.49–3.42; *p* = 0.605). Ninety-day mortality occurred in 7 patients (7.1%) in the OPEN group and 5 patients (5.1%) in the MIS group (OR 0.70, 95% CI 0.23–2.15; *p* = 0.531). Complete results are summarized in Table 3.

### Sensitivity analysis – primary outcomes

Sensitivity analysis was performed to further explore the data and presented above. The estimates remained materially unchanged after adjustment for BMI and tumour location. In the fully adjusted models, MIS was associated with lower odds of postoperative complications (OR 0.42, 95% CI 0.22–0.80) and a three-day shorter median hospital stay (median difference − 3 days, 95% CI -5.0 to -1.0), consistent with the primary analyses.

### Surgical and short-term pathological quality indicators

Median lymph node yield was 27 in the OPEN group and 23 in the MIS group. Quantile regression with bootstrap standard errors showed no statistically significant difference in median lymph node yield between groups (median difference − 7 nodes, 95% CI − 7 to 0, *p* = 0.143). Both groups exceeded accepted oncological adequacy thresholds. Additionally, R0 resection was achieved in 86.9 (*n* = 86/99) patients in the OPEN group and 90.9% (*n* = 90/99) patients in the MIS group. Logistic regression with robust standard errors demonstrated no statistically significant difference between groups (OR 1.57, 95% CI 0.60–4.12, *p* = 0.360).

Median blood loss was 100 mL in the OPEN group and 50 mL in the MIS group. Quantile regression with bootstrap standard errors demonstrated numerically lower intraoperative blood loss with MIS (median difference − 50 mL, 95% CI − 75 to − 0, *p* = 0.206).

Despite the technical challenges associated with emergency MIS in obstructed patients, duration of surgery was comparable between the two approaches at a median of 159 min in the OPEN group and 165 min in the MIS group (median difference + 6 min, 95% CI − 12 to 26, *p* = 0.456).

As perforated tumours constituted an exclusion criterion, no patients with primary tumour perforation were included in the study cohort. Intraoperative bowel perforation rates are presented in Table 2.

## Discussion

The present study compared MIS to OPEN emergency right-sided colectomy due to malignant obstruction. Current WSES guidelines do not provide recommendations regarding surgical approach in right-sided obstruction, highlighting an important knowledge gap addressed by this study [9].

MIS was associated with a clinically relevant reduction in length of stay of three days compared with OPEN surgery. Shorter LOS likely reflects a combination of fewer complications and faster postoperative recovery associated with MIS. However, LOS may also be influenced by institutional discharge practices and implementation of enhanced recovery protocols [20].

The present study included patients from a nationwide healthcare system encompassing a large number of hospitals, thereby reducing this potential source of bias. Together with the observed associations regarding both shorter LOS and lower postoperative complication rates, as well as the absence of increased readmission rates, the present findings support a potential beneficial association of MIS. Moreover, our results are consistent with those of Hussain et al. (2024), who also reported significantly shorter LOS after MIS in a comparable single-center study [4].

In emergency colorectal and general surgery with a variety of indications, MIS has previously been associated with shorter LOS, lower wound infection rates and decreased mortality [10–12, 21–24]. The present MIS cohort had a significantly lower overall complication rate, in line with previous studies reporting either reduced or similar complication rates in comparable settings [4, 11, 25]. Although point estimates favoured minimally invasive surgery for readmission and 90-day mortality, the present study was not powered to detect differences in infrequent outcomes. Consequently, the absence of statistically significant differences for mortality, readmission, reoperation and R0 resection should not be interpreted as evidence of equivalence between approaches.

Fewer postoperative complications might have contributed to shorter LOS in the MIS cohort. However, other advantages of MIS such as reduced postoperative pain, earlier return to normal diet and bowel function might also have impacted median LOS.

However, LOS may be influenced by factors not captured in the study, such as local hospital discharge practices and the implementation of fast-track pathways in emergency surgery. These can both affect LOS and complication rates [26]. Although the indication for surgery was bowel obstruction, information regarding the extent of obstruction (cecal or small bowel distention) was not available. Additionally, duration, severity of obstructive symptoms and physiological status beyond ASA-classification was not available. These limitations may have influenced both the choice of MIS as well as LOS. Consequently, residual confounding and selection bias cannot be excluded despite the use of propensity score matching.

Tumour location may also have influenced the choice of surgical approach. Lesions located at the hepatic flexure or proximal transverse colon can be technically more demanding because of their proximity to adjacent structures and the need for more extensive mobilization, whereas caecal and ascending colon tumours may be considered more favourable for a minimally invasive approach. Although tumour location was available and reported, the registry lacked detailed anatomical information regarding local tumour extent and adjacent organ involvement. Consequently, we could not determine whether such factors contributed to the observed selection of patients undergoing MIS.

No statistically significant differences were observed between MIS and OPEN for the available short-term surgical and pathological quality indicators, including surgical radicality, lymph node yield, intraoperative blood loss, and operative time. Operative duration may be affected by several factors that were not fully captured in the present study. Emergency surgery for obstructing right-sided colon cancer is often technically demanding because of bowel dilatation, impaired visualization, locally advanced tumour growth, adhesions, and the occasional need for multivisceral resection. Furthermore, surgeon experience and institutional familiarity with emergency minimally invasive colorectal surgery may substantially influence operative efficiency. This finding is consistent with previous studies suggesting that emergency MIS can be performed with operative duration of surgery comparable to open surgery when undertaken by experienced surgeons. Nevertheless, residual differences in procedural complexity may have influenced both operative duration and postoperative outcomes [1, 4, 27].

There was no difference in the proportion of permanent stoma creation as compared to primary anastomosis, which could be expected when matching for age and ASA-class. However, it is conceivable that other underlying baseline or urgent comorbidities influenced the surgeon’s decision to create a stoma.

According to SCRCR data for the study period, 58.0% of all elective resections were performed with MIS, compared with only 5.7% in the emergency setting [19]. The low proportion of MIS may reflect careful patient selection and concentration of emergency MIS expertise at selected centres, which may partly explain the favourable outcomes observed. MIS procedures were distributed across 31 hospitals, although a substantial proportion were performed in a limited number of centres, suggesting concentration of emergency MIS expertise. Previous studies have shown that MIS is feasible for colonic obstruction in selected patients when performed by experienced laparoscopic surgeons. However, no comparable national multicenter data have been published [21, 28]. It therefore remains unclear whether the low proportion of MIS in the present study reflects expected practice or underutilization. The relatively late introduction of elective MIS in Sweden has likely influenced its adoption in the emergency setting as well, suggesting that emergency MIS could potentially be more widely adopted [11].

Emergency surgery is associated with increased risk of complications and impaired oncological outcome. Current treatment guidelines therefore recommend avoiding emergency surgery whenever possible, particularly in locally advanced disease [9, 29]. However, in right-sided obstruction, decompression options are limited, and the role of self-expanding metal stents remains less well established [30]. European guidelines therefore offer primary emergency resection as an option in management of these patients, as compared with defunctioning stoma without resection, and without recommendation of a specific surgical approach [8, 9, 29, 31].

The study has several strengths, including the nationwide design, validated registry data, and use of propensity score matching. Limitations include the absence of physiological parameters, relatively low granularity of surgical outcomes, and a potential risk of selection bias despite clear inclusion criteria. The propensity score model was limited to variables available in the registry which may influence surgical approach. Important factors such as tumour burden, obstruction severity, and surgeon experience were not included, which may result in residual confounding and contribute to selection bias. Furthermore, it is important to note that the study is likely underpowered for less frequent outcomes such as re-admission and early mortality. Therefore, the observed associations may not be generalisable to the broader emergency population. Furthermore, since patients presenting with perforated colon cancer were excluded, our findings are not generalisable to emergency presentations complicated by perforation.

The decision to construct a stoma was made at the discretion of the operating surgeon, and no predefined criteria were applied within the study. As the registry does not record the specific indication for stoma formation, it was not possible to determine which clinical or intraoperative factors contributed to this decision. Consequently, unmeasured differences in factors influencing stoma formation may have existed between the groups. Additionally, surgeon-level data were unavailable, precluding adjustment for surgeon experience. Furthermore, the registry does not capture surgeon-specific information, operative volume, or institutional experience with emergency MIS. These factors may have influenced both treatment selection and outcomes and represent potential sources of residual confounding.

The principal limitation of the present study is residual confounding. Although propensity score matching improved comparability between groups for the available variables, several factors likely affecting both the choice of surgical approach and postoperative outcomes were unavailable. These include the severity and duration of obstruction, bowel distension, physiological status at presentation, tumour complexity, surgeon expertise, and institutional experience with emergency MIS. Consequently, patients selected for MIS may have differed from those undergoing open surgery in ways not captured by the registry, and the observed associations should therefore not be interpreted as causal effects.

Furthermore, only a minority of patients underwent an initially minimally invasive approach, suggesting substantial patient selection. The findings should therefore be interpreted as reflecting outcomes among carefully selected patients deemed suitable for MIS rather than the broader population of patients undergoing emergency right-sided colectomy for malignant obstruction.

Despite these limitations, the demonstrated feasibility of MIS supports further evaluation and careful expansion in emergency patients. However, identifying which patients truly benefit from emergency MIS and those who may be at risk of harm was beyond the scope of this study, and requires further evaluation to better guide this choice. Future research should assess long-term oncological outcomes of MIS in emergencies, incorporate patient-reported outcomes, and ideally be conducted prospectively. Such a study is currently underway in Finland [5].

### Clinical implications

- MIS may be feasible in carefully selected emergency patients
- Surgeon experience is likely critical
- Patient selection remains key
- Not generalizable to all emergency settings

## Conclusion

In this nationwide observational cohort, an initially minimally invasive approach to emergency right-sided colectomy for malignant obstruction was associated with shorter length of stay and fewer recorded postoperative complications. No statistically significant differences were observed in the available short-term surgical and pathological quality indicators. Given the marked patient selection, limited number of MIS procedures and potential for residual confounding, these findings support the feasibility of MIS in carefully selected patients with right-sided malignant colonic obstruction.

## Supplementary Information

Supplementary Material 1.

Supplementary Material 2.

Supplementary Material 3.

## Data Availability

All data used is available through the Swedish Colorectal Cancer Registry. The datasets analysed are not publicly available due to institutional and ethical restrictions but are available from the corresponding author on reasonable request and with permission from the relevant authorities.

## Abbreviations

ASA: American Society of Anesthesiologists
BMI: Body mass index
CI: Confidence interval
CT: Computed tomography
IQR: Interquartile range
ITT: Intention-to-treat
LOS: Length of stay
MIS: Minimally invasive surgery
OPEN: Open surgery
OR: Odds ratio
PSM: Propensity score matching
SCRCR: Swedish Colorectal Cancer Registry
SD: Standard deviation
SMD: Standardized mean difference
WJES: World Journal of Emergency Surgery
