# Supplementary material for: Minimally invasive versus open emergency right-sided colectomy for malignant obstruction: a nationwide propensity score–matched cohort study
Source: World J Emerg Surg. 2026 Oct 6;21:54. doi: 10.1186/s13017-026-00735-6 (PMC13640095; doi:10.1186/s13017-026-00735-6)
Supplement: Supplementary file 2 [file 13017_2026_735_MOESM2_ESM.docx]

| Centre | Total | OPEN | MIS |
| --- | --- | --- | --- |
| I | 21 | 13 | 8 |
| II | 17 | 6 | 11 |
| III | 12 | 5 | 7 |
| IV | 11 | 4 | 7 |
| V | 11 | 5 | 6 |
| VI | 9 | 0 | 9 |
| VII | 9 | 5 | 4 |
| VIII | 8 | 1 | 7 |
| IX | 7 | 4 | 3 |
| X | 6 | 3 | 3 |
| XI | 6 | 5 | 1 |
| XII | 5 | 0 | 5 |
| XIII | 5 | 5 | 0 |
| XIV | 4 | 2 | 2 |
| XV | 4 | 2 | 2 |
| XVI | 4 | 3 | 1 |
| XVII | 4 | 2 | 2 |
| XVIII | 4 | 1 | 3 |
| XIX | 4 | 4 | 0 |
| XX | 4 | 1 | 3 |
| XXI | 3 | 2 | 1 |
| XXII | 3 | 2 | 1 |
| XXIII | 3 | 1 | 2 |
| XXIV | 2 | 1 | 1 |
| XXV | 2 | 0 | 2 |
| XXVI | 2 | 2 | 0 |
| XXVII | 2 | 2 | 0 |
| XXVIII | 2 | 1 | 1 |
| XXIX | 2 | 2 | 0 |
| XXX | 2 | 2 | 0 |
| XXXI | 2 | 2 | 0 |
| XXXII | 2 | 0 | 2 |
| XXXIII | 2 | 1 | 1 |
| XXXIV | 2 | 2 | 0 |
| XXXV | 1 | 0 | 1 |
| XXXVI | 1 | 1 | 0 |
| XXXVII | 1 | 1 | 0 |
| XXXVIII | 1 | 0 | 1 |
| XXXIX | 1 | 1 | 0 |
| XL | 1 | 1 | 0 |
| XLI | 1 | 0 | 1 |
| XLII | 1 | 1 | 0 |
| XLIII | 1 | 1 | 0 |
| XLIV | 1 | 0 | 1 |
| XLV | 1 | 1 | 0 |
| XLVI | 1 | 1 | 0 |

**Supplement 2. Distribution of operative approach across centres**

Values are *n* of patients in the matched cohort.
