# Supplementary material for: Minimally invasive versus open emergency right-sided colectomy for malignant obstruction: a nationwide propensity score–matched cohort study
Source: World J Emerg Surg. 2026 Oct 6;21:54. doi: 10.1186/s13017-026-00735-6 (PMC13640095; doi:10.1186/s13017-026-00735-6)
Supplement: Supplementary file 3 [file 13017_2026_735_MOESM3_ESM.docx]

**STROBE Checklist**

Manuscript title: Minimally invasive versus open emergency right-sided colectomy for malignant obstruction: a nationwide propensity score–matched cohort study

| **Section** | **Item** | **STROBE recommendation** | **Page/section in manuscript** |
| --- | --- | --- | --- |
| Title and abstract | 1a | Indicate study design in title | Title |
|  | 1b | Provide informative abstract | Abstract |
| Introduction | 2 | Background and rationale | Introduction |
|  | 3 | Objectives and hypotheses | End of Introduction |
| Methods | 4 | Study design | Methods |
|  | 5 | Setting | Methods |
|  | 6a | Eligibility criteria | Methods |
|  | 6b | Matching criteria | Statistical analysis, Table1, Suppl. fig.1 |
|  | 7 | Variables defined | Methods |
|  | 8 | Data sources | Methods |
|  | 9 | Bias addressed | Methods; Discussion (Limitations) |
|  | 10 | Study size | Statistical analysis; Discussion (Limitation) |
|  | 11 | Quantitative variables | Methods |
|  | 12 | Statistical methods | Statistical analysis; Table 1; Suppl Fig 1 |
| Results | 13 | Participants flow | Figure 1; Results (Study population) |
|  | 14 | Descriptive data | Table 1; Results (Study population) |
|  | 15 | Outcome data | Table 2-3; Results |
|  | 16 | Main results | Results; Table 3 |
|  | 17 | Other analyses | Results; Suppl fig. 1 |
| Discussion | 18 | Key results | Discussion |
|  | 19 | Limitations | Discussion |
|  | 20 | Interpretation | Discussion |
|  | 21 | Generalizability | Discussion |
| Other | 22 | Funding | Declarations |
