# Supplementary material for: Minimally invasive versus open emergency right-sided colectomy for malignant obstruction: a nationwide propensity score–matched cohort study
Source: World J Emerg Surg. 2026 Oct 6;21:54. doi: 10.1186/s13017-026-00735-6 (PMC13640095; doi:10.1186/s13017-026-00735-6)
Supplement: Supplementary file 4 [file 13017_2026_735_MOESM1_ESM.docx]

**Supplement 1. Covariate balance before and after matching**
